# Surgery of the Spinal Cord

**Published:** 1907-04

**Authors:** 


### SURGERY OF THE SPINAL CORD.

Dr. Jno. B. Murphy discusses this subject with special reference to the points in the diagnosis and the definite indication for surgical treatment in the cases amenable to it, and presents the following conclusions:

The gray and white matter of the spinal cord down to the cauda is made up of:

(a) Intrinsic ganglionic cells (gray matter).

(b) Centrifugal cerebral axons (white matter of motor columns).

(c) Centripetal sensory axons from the spinal ganglia in which is located the trophic cell of the body of these axons, making up the posterior sensory column. The centripetal and centrifugal axons within the spinal cord are aneurilemmic.

(d) Intercommunicating or associating axons which connect the various segments of the intrinsic ganglionic cells of the cord, and form the anterior ground bundle of white fibers.

(c) Direct cerebellar centripetal axons originate from ganglionic cells situated in the posterior internal region or posterior horn and close to the commissure, leading to the ganglionic cells situated in the vermis superior of the cerebellum and are aneurilemmic (tract of equilibrium).

(7) Gowers' or the lateral spinal tract, the axons of which originate in the posterior horn, close to the commissure.

(g) Neuroglia.

(h) The bulb or medulla oblongata is a continuation of the same ganglionic zones of gray matter in the cord and has all of the centrifugal and centripetal axons of the spinal cord. In addition, it has its own peculiar anatomic elements.

Nerves arising from the medulla are surgically spinal nerves. Therefore, all of the "cranial" nerves, except those of special sense, are from a surgical standpoint, essentially spinal nerves. The extracordal motor and sensory axons are medullated and neurilemmic.

(f) Nerves of special sense should be called cerebral nerves, as their trophic cell bodies are outside the cranium, and in the organs of special sense. The centripetal axons of these nerves, with the exception of the olfactory, are all medullated and all aneurilemmic.

The extra spinal root, both centrifugal and centripetal sensory, are medullated and neurilemmic. The peripheral nerves of the bulb and cord (cranial and spinal) are medullated and neurilemmic to their nerve endings. The terminal dendrites are medullated and aneurilemmic. They possess, however, a limited potentiality of outgrowth of the axons beyond their neurilemma. The caudia, from a surgical standpoint, is an intradural collection of extramedullary spinal axons, and is made up of axons with a medullary sheath and neurilemma. The sympathetic system is a chain of ganglia and nerve trunks. Their axons are neurilemmic and non-medullated.

When an axon is divided, the entire distal portion from the trophic ganglionic body degenerates; proximally, it degenerates for a short distance, and for a longer distance it atrophies. A ganglion cell once destroyed, the degeneration of its axon, or axons and dendrites is complete, and the restoration and sensation in its peripheral zone can only be obtained by anastomosis of its axons to other axons originating from healthy ganglonic cells, as in anterior poliomyelitis.

Ganglionic cells and aneurilemmic axons once destroyed never regenerate. All neurilemmic axons, centrifugal (motor), and centripetal (sensory), are capable or potent of both anatomic and physiologic regeneration under favorable conditions. For the role played by the neurilemma in nerve regeneration the reader is referred to the article on regeneration of peripheral nerves. Therefore,

(a) Ganglionic cells in the gray matter of the cord never regenerate. (b) Centrifugal motor axons within the cord never regenerate, as they are aneurilemmic. (c) Centripetal sensory axons within the cord never regenerate, as they are aneurilemmic.

It can, therefore, be seen that there is no regeneration of the white columns or gray matter of the cord after destruction or division; it has never been demonstrated experimentally nor authoritatively observed clinically. All these principles apply equally to the bulb or medulla oblongata. The spinal, cranial, sensory and sympathetic ganglions once destroyed never regenerate. The caudal portion of the cord is made up of neurilemmic centrifugal axons from the spinal cord ganglion cells, in the conus, and of centripetal sensory axons from cells in the spinal ganglia, which are situated extra dural in the lumbar and sacral foramina, both are neurilemmic, from the place they leave or enter the conus. They are, therefore, all capable of regeneration, the same as peripheral axons.

The extracordal or extramedullary and extrabulbous spinal and cranial axons are neurilemmic and potent of regeneration. Therefore, divided roots within the cranium and spinal canal regenerate. Peripheral nerves, cranial and cerebral, are composed of neurilemmic axons and capable of regeneration. The nerves of special sense, the optic, olfactory, gustatory and auditory, are composed of aneurilemmic axons, and are incapable of regeneration if once destroyed. In this particular, they resemble the columns of the cord. Their trophic cells are situated in the retina, Schneiderian mucosa, taste organs, cochlea and vestibule. When these membranes and ganglion cells are destroyed, the cells and axons are incapable of regeneration. The sympathetic nerve trunks are non-medullated, but neurilemmic and capable of regeneration.

Hemorrhage, concussion and contusion without laceration may offer the same immediate symptomatic picture as that of division, and a positive differential diagnosis may be practically impossible.

There is no direct relationship between the severity of the trauma and the degree of lesion in the cord. The element of time and the order of appearance of symptoms is of great importance, and may be the only guide in the differential diagnosis.

Absence of paralytic symptoms immediately after spinal trauma does not justify the surgeon in assuring the patient that such symptoms will not appear. They may set in within a few days or even some weeks after the injury.

If paralysis is due to hematorrachis the condition can be relieved by early spinal puncture. Intra or periarachnoidal hemorrhage from bullet or stab wound may produce complete paralysis resembling a division of the cord. If the pressure be relieved the patient will survive and paralysis will be temporary.

The majority of cases of transverse or incomplete traumatic irregular paralysis following fractures recovers without operative treatment, which signifies an absence of spinal cord division. Wherever there is immediate and complete circular paralysis, operation does not benefit the patient in the least, as there has been a division of spinal axons which never regenerate.

Surgical intervention in injuries to the cord should be resorted to only in cases where the spinal cord is not completely divided, except in the caudal zone. Immediate intervention will be of benefit when the cord is compressed above the cauda, or compressed and divided in the cauda. If operation is at all indicated there is no reason for delay, as degeneration changes may take place in the cells and neurons of the cord, which would be as irreparable as its division.

In fractures of the spine without considerable displacement, we are justified in assuming that the cord is not suffering continual compression, regardless of the degree of paralysis, and operation is contraindicated. If this paralysis is due to laceration, it will not be improved by operation. If it is due to contusion it will recover without operation.

In gunshot and stab wounds with immediate paralysis, operation is contraindicated, as the cord is probably severed, and its re-approximation will avail nothing, except in the caudal zone. After division or crushing of the nerves of the caudal zone, there is a positive indication for an end-to-end suture, axonal contact of the various fibers, as determined by faradic stimulation, the same as in peripheral nerves.

In spina bifida centralis paralytica, in the caudal zone, resection of the atrophied section of the spinal cord with end-to-end union is indicated. Apendymal spina bifida should be treated by resection of compressed segments, and accurate suture approximation of the fasciculi of the cauda. Upper ependymal and true cord central spina bifida may be treated by ependymal subarachnoidal drainage.

In all non-malignant tumors of the cord, laminectomy should be performed at the onset of paresis. Delay to complete paralysis is unpardonable, not to use a more forcible expression. Operation after complete paralysis from compression with degeneration is contraindicated.

In tuberculoma compression the spinal cord operations should be done at the beginning of symptoms of paralysis. Late operations, that is, after a pressure necrosis of the cord, are worthless. The bone cavity should be filled with a Moorhof plug, as this lessens the liability of suppuration and hastens the process of repair.

In conclusion, surgery of the spinal cord, like surgery of other parts of the body, must be timely, i. e., operation must be done before the pathologic condition is advanced beyond the possibility of repair. In our present positive, though limited knowledge, timely action means conservatism, while delay must be interpreted as timidity and inefficiency.—Jour. A. M. A., March 2, '07.

